# Comparative molecular dynamics of two cyclic peptide inhibitors bound to the Zika virus NS2B/NS3 protease

**DOI:** 10.1007/s00894-026-06945-8

**Published:** 2026-09-07

**Authors:** Camilla Vitoria Silva Marinho, Maycon Vinicius Damasceno de Oliveira, Anderson H. Lima

**Affiliations:** Laboratório de Planejamento e Desenvolvimento de Fármacos, Instituto de Ciências Exatas e Naturais, Universidade Federal do Pará, Belém, Pará, 66075-110 Brazil

**Keywords:** ZIKV protease, Macrocyclic peptides, Conformational preorganization, Binding free energy

## Abstract

**Context:**

The Zika virus NS2B/NS3 protease is an important antiviral target, and macrocyclic peptide inhibitors represent promising scaffolds because they combine multibasic recognition motifs with conformational restriction. Here, we investigated two structurally related cyclic peptide inhibitors, referred to as CP1 and CP2, which share a conserved macrocyclic framework but differ in the chemical nature of their linker substituents. Molecular dynamics simulations showed that both peptides display broad intramolecular distance distributions in aqueous solution, indicating that key crystallographic contacts are not intrinsically maintained in the unbound state. Upon binding to the protease, CP2 adopted a more defined conformational ensemble and preserved a short intramolecular contact compatible with its crystallographic arrangement, whereas CP1 lost its crystallographic d2 contact and sampled a broader bound-state distribution. CP2 also showed a more focused interaction network involving residues from the catalytic and substrate-recognition regions and more favorable binding free-energy estimates.

**Methods:**

Molecular dynamics simulations were performed for each cyclic peptide in aqueous solution and bound to the Zika virus NS2B/NS3 protease using Amber 20. The protein was described with the ff14SB force field, the cyclic peptides with GAFF2, and the systems were solvated with the TIP3P water model. Three independent 500 ns simulations were performed for each system. Trajectories were analyzed using structural clustering, intramolecular distance distributions, residue-level interaction analysis, and MM/PBSA and MM/GBSA binding free-energy calculations.

**Supplementary Information:**

The online version contains supplementary material available at https://doi.org/10.1007/s00894-026-06945-8.

## Introduction

Zika virus (ZIKV) is an arbovirus, i.e., a virus transmitted by arthropods capable of infecting vertebrate hosts [1]. Taxonomically, ZIKV belongs to the family *Flaviviridae* and the genus *Flavivirus*, whose members share important structural and genomic features, including a positive-sense single-stranded RNA genome [2, 3]. Among flaviviruses, ZIKV is relevant not only because of its epidemiological impact but also because of the diversity of its transmission routes. In addition to vector-borne spread, ZIKV may also be transmitted vertically, from mother to fetus, sexually, and through blood transfusion [4, 5]. Besides that, ZIKV infection is especially concerning because of its marked association with neurological complications [6–8]. Its ability to reach the amniotic environment has been associated with severe adverse pregnancy outcomes, including microcephaly, spontaneous abortion, intrauterine fetal death, impaired fetal growth, and placental dysfunction [6]. In addition, ZIKV infection has been linked to Guillain-Barré syndrome, a neurological disorder characterized by demyelination that may result in paralysis [7]. Together, these features reinforce the need for effective therapeutic strategies against ZIKV infection [8].

To date, no specific therapy has been approved for the prevention or treatment of ZIKV infection [9]. In this context, the search for antiviral agents has partly relied on drug repurposing strategies involving compounds already approved by regulatory agencies such as the Food and Drug Administration (FDA), with the aim of accelerating the development of therapeutic alternatives. However, the development of new antivirals remains challenging, since candidate molecules must combine antiviral efficacy with low cytotoxicity and minimal impact on host cell viability [10].

In this context, cyclic peptides have attracted particular attention because cyclization can enhance conformational stability, improve resistance to proteolytic degradation, and, in some cases, favor membrane permeability [11, 12]; due to their ability to adopt different conformations, adjustments in peptide structures may occur to improve cell membrane permeability without necessarily acquiring the conformation observed in their crystalline structure. The ZIKV NS2B/NS3 protease is an especially relevant antiviral target because it mediates cleavage of the viral polyprotein into functional nonstructural proteins required for viral replication. This enzyme comprises the N-terminal serine protease domain of NS3, which contains the catalytic triad His51-Asp75-Ser135, and the NS2B cofactor, which is essential for proper folding, substrate recognition, and catalytic activity [13]. Previous studies on ZIKV NS2B/NS3 protease inhibitors have shown that basic motifs within macrocyclic scaffolds may contribute to antiviral efficacy [14]. In particular, Huber et al. investigated a series of macrocyclic inhibitors sharing a common core structure but differing in the hydrophobic character of the linker region, demonstrating that these modifications can influence both active-site recognition and physicochemical properties relevant to biological activity, such as conformational stability and permeability [15].

Despite these advances, the dynamic basis by which subtle structural modifications alter the behavior of macrocyclic inhibitors in solution and in the protease-bound state remains insufficiently understood. In particular, understanding how linker-dependent conformational preferences affect intramolecular stabilization, protein-ligand recognition, and binding energetics may provide valuable insight into the structure-activity relationships of these compounds.

**Fig. 1:**
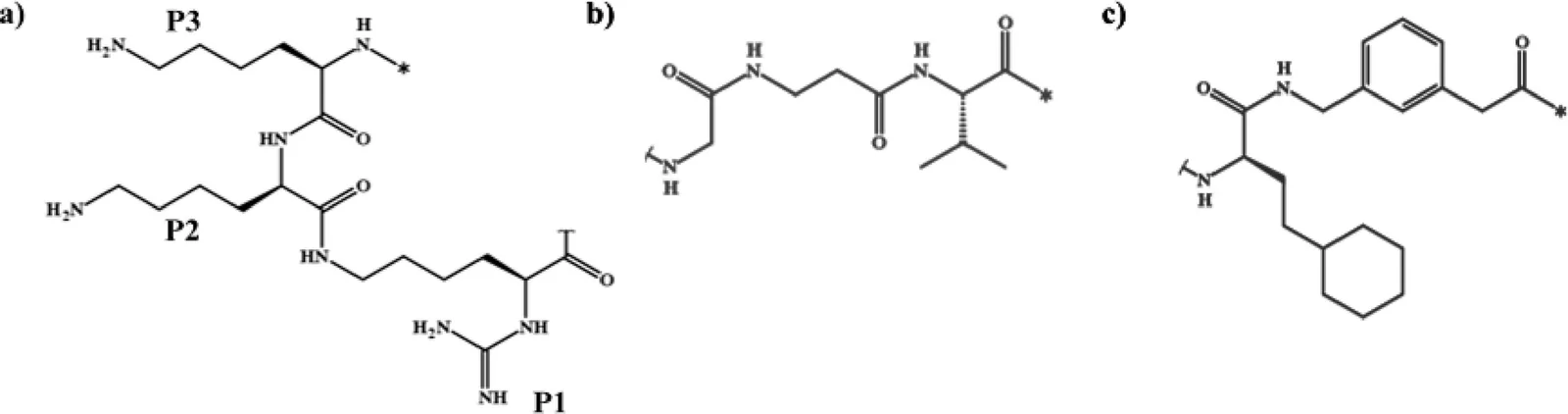
Two-dimensional representation of the cyclic peptide inhibitors. **a** Common macrocyclic core conserved in both compounds, **b** structural fragment corresponding to the linker region of CP1, and **c** structural fragment corresponding to the linker region of CP2. The wavy bonds and asterisks indicate the attachment points through which each substituent is connected to the common cyclic scaffold

Based on this premise, the present study investigated two macrocyclic peptide inhibitors originally reported by Huber et al.: compound 15, hereafter referred to as cyclic peptide 1 (CP1), which contains an L-valine residue in the linker region, and compound 26, hereafter referred to as cyclic peptide 2 (CP2), which contains a more hydrophobic D-homocyclohexylalanine residue in the linker segment. These two peptides share the same general macrocyclic inhibitor (Fig. 1) scaffold but differ in the chemical nature and hydrophobic character of the linker substituent.

We investigated how these linker differences influence the conformational ensembles of the isolated peptides in aqueous solution, their reorganization within the ZIKV NS2B/NS3 protease binding site, and the resulting protein-ligand interaction patterns. The objective was not to derive a quantitative structure-activity relationship for the complete inhibitor series, but to obtain an atomistic description of how two contrasting linker architectures affect the free and protein-bound conformational states of this class of macrocyclic peptides, offering theoretical support for the rational design of improved inhibitors targeting the ZIKV protease.

## Methodology

### Preparation of the ZIKV NS2B/NS3 protease and cyclic peptides

The structural models of the ZIKV NS2B/NS3 protease in complex with the cyclic peptide inhibitors were obtained from the Protein Data Bank (PDB) [16]. Specifically, the complex containing compound 15, hereafter referred to as CP1, was obtained from the structure with PDB ID 7PFY, whereas the complex containing compound 26, hereafter referred to as CP2, was obtained from the structure with PDB ID 7PGC. The crystallographic coordinates of CP1 and CP2 were retained as the initial binding poses for the protein-ligand molecular dynamics simulations.

Prior to molecular dynamics simulations, the protein structures were prepared by assigning protonation states at pH 7.4 using the PDB2PQR module available through the Adaptive Poisson-Boltzmann Solver (APBS) server [17]. His51 was modeled as the HID tautomer, with Nδ1 protonated and Nε2, which is oriented toward Ser135, unprotonated. Missing hydrogen atoms were added to the cyclic peptide inhibitors using UCSF Chimera [18]. For each inhibitor, a single conformation derived from its crystallographic complex was used for charge derivation. The structures were subjected to optimization in Gaussian09 [19] at the Hartree-Fock level [20] using the 6-31G* basis set. The electrostatic potential calculated from the resulting geometry was used to derive atomic partial charges through the Restrained Electrostatic Potential method [21]. GAFF2 atom types [22] and parameters were assigned to the complete macrocyclic structures, and missing bond, angle, and dihedral parameters were identified and generated using Parmchk2.

### System construction

System construction and topology generation were performed with the tLEaP module of the Amber 20 package. For each cyclic peptide, two simulation environments were built in order to evaluate both its intrinsic conformational behavior and its protease-bound behavior. In the first set of systems, the peptide was simulated in aqueous solution, containing only the ligand, explicit water molecules, and counterions. The peptides were parameterized with GAFF2 [22] and solvated in a cubic water box using the TIP3P water model [23]. In the second set, the peptide was placed in complex with the ZIKV NS2B/NS3 protease, generating the corresponding protein-peptide system, where the protein was described with the ff14SB force field [24]. All systems were solvated in explicit cubic water boxes using the TIP3P model, with a minimum distance of 12 Å between the solute and the box edges. Ions were added to neutralize the net charge of each system [25].

Before the molecular dynamic simulations, each system was subjected to a multistep energy minimization protocol designed to remove unfavorable steric contacts introduced during system assembly and solvation. For all systems, the first minimization stage consisted of 4000 cycles applied to water molecules and peptide hydrogen atoms, while the remaining atoms were restrained. This initial step was intended to relax the solvent environment and local hydrogen positions without significantly perturbing the starting solute geometry.

All systems were gradually heated from 10 to 298 K over 200 ps using a Langevin thermostat. Following heating, a 300 ps density-adjustment run was performed to allow solvent reorganization around the solute. The systems were then equilibrated for 500 ps under isothermal-isobaric conditions at 298 K and 1 atm using the Berendsen barostat.

Production MD simulations were carried out in triplicate for each system in order to assess the reproducibility of the observed conformational behavior. For each replica, a 500 ns trajectory was generated, resulting in three independent production runs per aqueous system and three per protein-bound system. During the simulations, nonbonded interaction cutoffs of 8 Å were used for the aqueous peptide systems, whereas a cutoff of 10 Å was employed for the protein-bound systems. The integration time step was set to 2 fs, and the equations of motion were propagated using the Verlet algorithm [26].

### Structural analysis and clustering

Root mean square deviation (RMSD) and root mean square fluctuation (RMSF) analyses were performed separately for each independent production trajectory. Prior to the calculations, periodicity was removed and each trajectory frame was least-squares fitted to the minimized starting structure using the backbone atoms of the protease. RMSD values were calculated separately for the NS2B and NS3 regions and for the cyclic peptide. Protein RMSF values were calculated for the Cα atoms after the same protein-based alignment.

For each protein-peptide complex, the trajectories from the three independent replicas were combined into a single 1.5 µs trajectory for clustering analysis. A refinement clustering algorithm based on the *k-means* method was applied using the RMSD of the complex backbone atoms as the similarity metric [27]. Five clusters and a maximum of 500 iterations were used. The representative structure of each cluster was defined as the structure with the minimum sum of squared displacements relative to the other structures assigned to the same cluster.

### Define Secondary Structure of Proteins (DSSP)

Secondary-structure assignments were calculated using the Define Secondary Structure of Proteins method [28] for every frame of each 500 ns production trajectory. The analysis was performed separately for each independent replica, and the reported populations correspond to the mean values obtained from the three replicas. Particular attention was given to the flexible NS2B segment located between β-strands β1 and β2, which was identified from the residue-wise RMSF profiles. Whole-protease flexibility was independently evaluated using RMSF calculations for the complete NS2B and NS3 regions.

### Binding free-energy calculations

To estimate the energetic favorability of complex formation between the cyclic peptides and the ZIKV NS2B/NS3 protease, end-point free-energy methods were applied to the production trajectories, namely MM/PBSA and MM/GBSA [29–31]. In these approaches, the total energetic contribution is decomposed into molecular mechanics and solvation terms. The molecular mechanics contribution, Δ*E*_MM_, comprises internal, electrostatic, and van der Waals components, as shown in Eq. 1.

1 $$\Delta {E}_{MM} = \Delta {E}_{int} + \Delta {E}_{ele} + \Delta {E}_{vdW}$$

The solvation contribution, Δ*G*_sol_, includes polar and nonpolar terms, as shown in Eq. 2.

2 $$\Delta {G}_{sol} = \Delta {G}_{pl} + \Delta {G}_{np}$$

In the MM/PBSA calculations, the polar contribution was evaluated by solving the Poisson-Boltzmann equation, whereas in the MM/GBSA calculations, it was estimated using the Generalized Born approximation. The nonpolar solvation term was associated mainly with solvent-accessible surface area and hydrophobic solute-solvent interactions. The total free energy was then obtained according to Eq. 3.

3 $$\Delta {G}_{total} = \Delta {E}_{MM} + \Delta {G}_{sol}$$

The polar solvation contribution was evaluated using the modified Generalized Born model of Onufriev, Bashford, and Case implemented in AMBER as igb = 2. The model employed the empirical parameters *α* = 0.8, *β* = 0.0, and *γ* = 2.909125.

MM/PBSA was calculated using an optimization of the atomic radii through the asymmetric Generalized Born hydration charge model (CHAGB), in which the dielectric surface, defined from the atomic radii present in the topology, is shifted outward relative to the molecular surface. In both models, a salt concentration of 0.100 mol L^−1^ was considered.

To include the entropic contribution, a normal mode analysis was performed. Thus, the entropy-corrected binding free energy was calculated according to Eq. 4:

4 $${\Delta G}_{bind}={\Delta G}_{MM/GBSA}-{T\Delta S}_{NMA}$$

For each replica, free-energy calculations were performed over 50,000 frames extracted from the 500 ns production trajectories, with trajectory sampling carried out every 5 frames, the set of atoms used consisted of all atoms from the peptides and proteases, excluding water molecules and counterions. This analysis was used to compare the relative energetic stability of the two peptide-protease complexes and to support the interpretation of the structural and conformational results obtained from the MD simulations. Per-residue energetic decomposition was performed separately for each independent replica. For each residue, the value presented corresponds to the mean of the three replica averages, and the error bars represent the standard deviation among the independent replicas.

The solvated interaction energy was estimated using SIETRAJ. In this approach, the interaction energy calculated with the AMBER force field is combined with the energetic cost of ligand and protein desolvation [32, 33]. Computational alanine scanning was subsequently performed for selected residues surrounding the peptide-binding site. For each mutation, the energetic effect was calculated according to Eq. 5:

5 $$\Delta \Delta {G}_{Ala} = {\Delta G}_{bind}^{Ala}- {\Delta G}_{bind}^{WT}$$

Positive $\Delta \Delta {G}_{Ala}$ values therefore indicate that replacement by alanine destabilizes the complex and that the original residue contributes favorably to binding. The analysis was performed separately for each simulation replica, with energies evaluated every five trajectory frames. The reported values correspond to the mean ± standard deviation across the three independent replicas.

## Results and discussion

### Overall conformational stability

The present work investigates the structural behavior of cyclic peptide inhibitors targeting the ZIKV NS2B/NS3 protease through molecular dynamics simulations. The two ligands examined here share the same tribasic macrocyclic recognition framework (Fig. 1) but differ in the architecture of the linker region. CP1 corresponds to compound 15 reported by Huber et al. [15], a P4 L-valine derivative whose altered linker conformation was associated with reduced stabilization and lower inhibitory potency, whereas CP2 corresponds to compound 26, which contains a 3-aminomethylphenylacetyl linker segment followed by a D-homocyclohexylalanine residue adjacent to P1 and was identified as one of the most potent compounds in that series. Consistent with these structural differences, Huber et al. reported a markedly weaker activity for compound CP1 than for compound CP2, with CP2 displaying a *K*_i_ of 2.33 nM against the ZIKV protease, while compound CP2 showed substantially lower potency (340 nM). These experimental results suggest that changes in linker composition can strongly influence how the macrocycle is organized and recognized by the protease, providing the basis for the comparative dynamics analysis performed here.

**Fig. 2:**
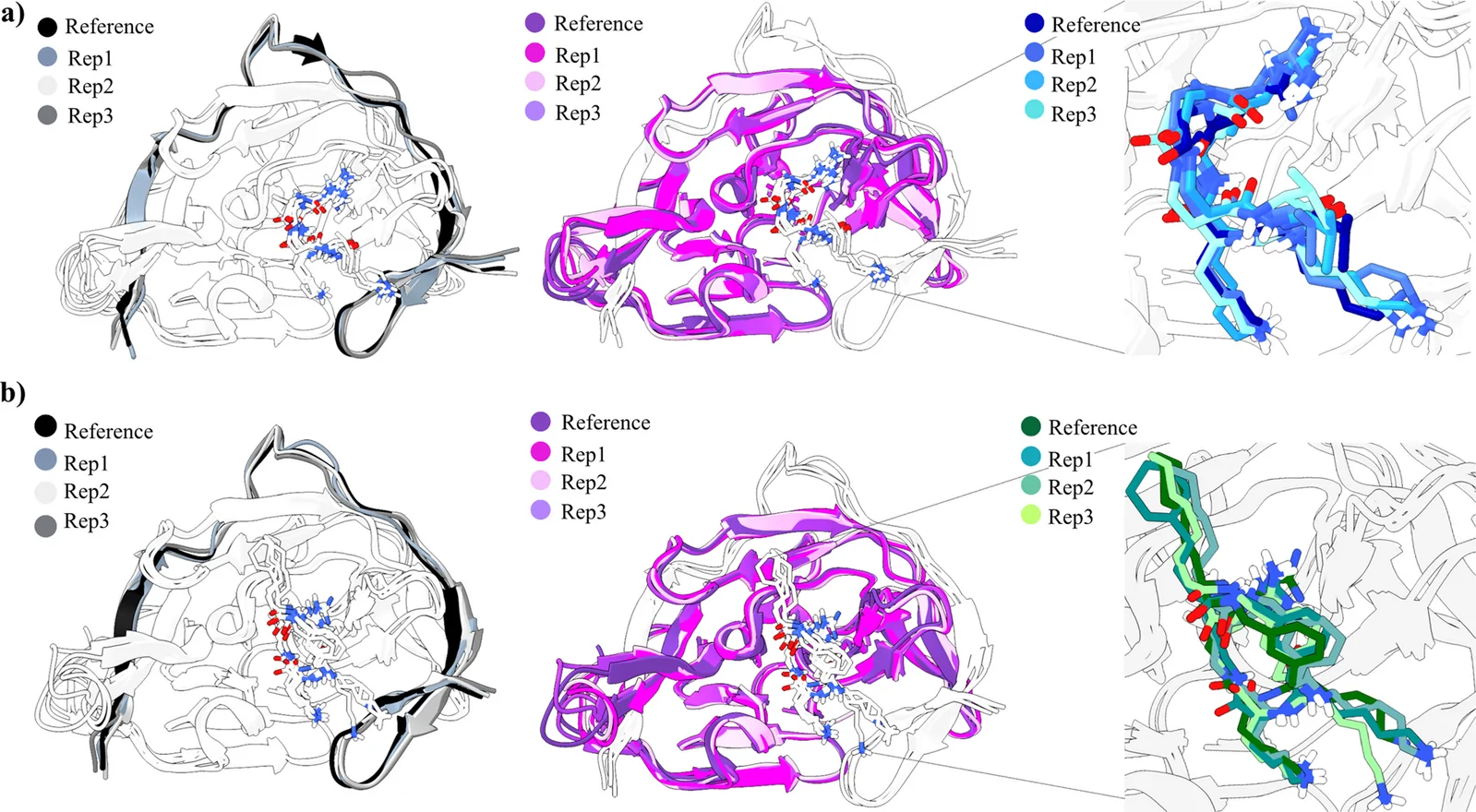
Reproducibility of the MD replicas and ligand-dependent bound conformations of the ZIKV NS2B/NS3 protease complexes. **a** Complex containing CP1 and **b** complex containing CP2. Representative structures from the most populated cluster of the three independent replicas are superposed. The close overlap among replicas indicates reproducible sampling for both systems. The structure indicated in the caption as the reference corresponds to the crystallographic structure of each complex

To assess whether these structural differences are reflected in the dynamic behavior of the complexes, we first examined the reproducibility of the MD simulations by comparing representative structures from the most populated cluster of each independent replica (Fig. 2). The results indicate that the trajectories converged toward closely related bound states rather than replica-specific conformations. In both complexes, the overall fold of the ZIKV NS2B/NS3 protease was preserved [34, 35]. It is important to mention that the use of multiple independent replicas is particularly important for cyclic peptides, whose conformational landscapes can be rugged and highly sensitive to local geometric restraints [36].

The RMSD profiles of the individual replicas further support the overall structural stability of both complexes (Figs. S1 and S2). The protein and peptide RMSD values fluctuated around approximately stable plateaus, without evidence of progressive structural drift, global unfolding, or ligand dissociation. Replica 3 showed larger conformational excursions, particularly in the NS2B region, but retained the global NS2B/NS3 fold and the peptide within the binding site. Consistently, the RMSF profiles localized the largest fluctuations mainly to the flexible NS2B segment between β-strands β1 and β2, whereas most of the NS3 protease domain remained comparatively less flexible.

Despite this common global behavior, the two ligands occupied the binding region differently. CP1 remained more centrally confined within the catalytic cleft, whereas CP2 sampled a broader interaction footprint and extended more clearly toward adjacent surface regions (see Fig. S3). This observation is consistent with the crystallographic analysis of Huber et al., who showed that compound CP1 adopts a linker arrangement that is less effectively stabilized, while CP2 preserves a favorable macrocyclic geometry and gains additional van der Waals contacts with the protease.

**Fig. 3:**
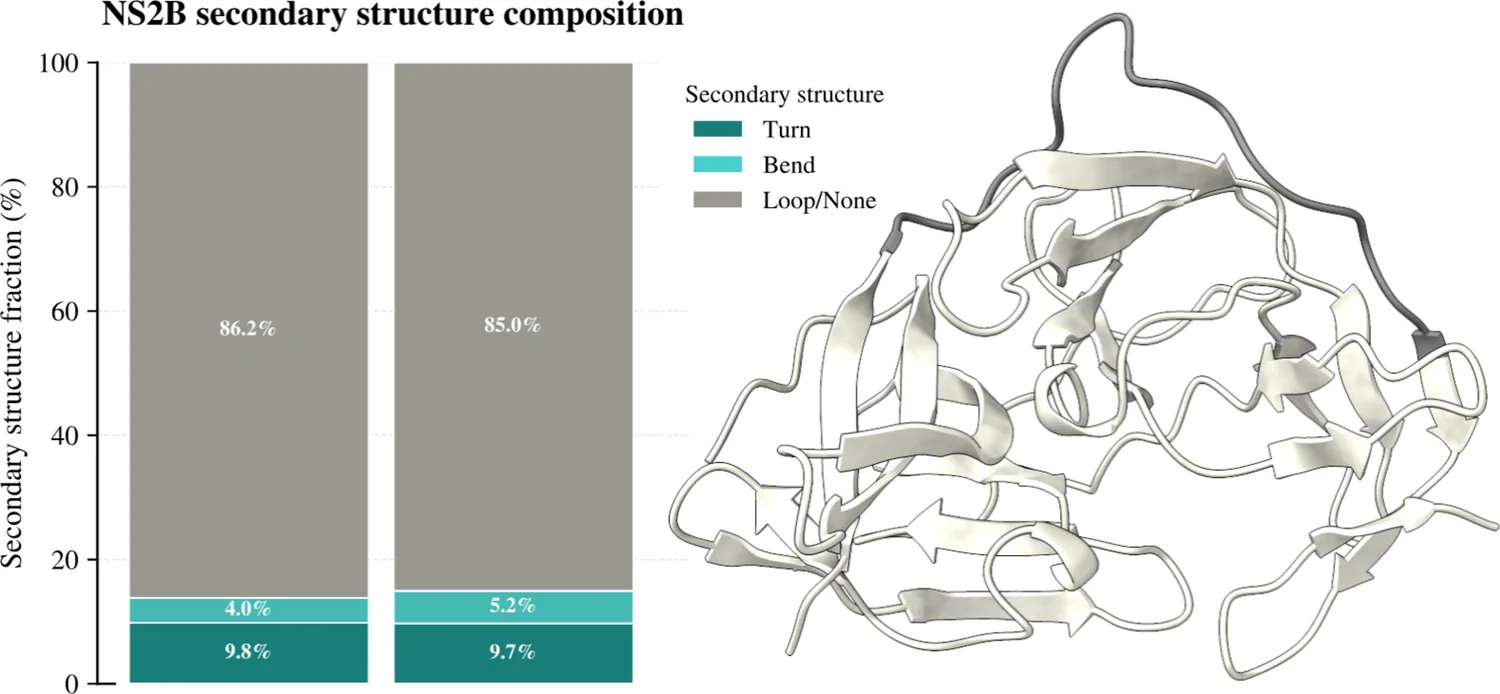
DSSP-derived secondary-structure populations of the selected NS2B segment between β-strands β1 and β2 in the CP1- and CP2-bound ZIKV NS2B/NS3 protease systems. Values correspond to averages obtained from the three independent 500 ns replicas

In both complexes, loop was the dominant secondary-structure element, indicating that this NS2B region preserves a locally organized conformation during the simulations (Fig. 3).

However, the distribution differed between the two systems. In the complex with inhibitor CP1, the NS2B segment showed 9.8% turn and 4.0% bend, whereas in the complex with CP2, the turn population decreased to 9.7%, with a corresponding increase in bend content to 5.2%. This result suggests that both CP1 and CP2 stabilize turn-like arrangement of this NS2B region, while CP2 allows a higher bend population, indicating greater local conformational plasticity. Since this NS2B segment contributes to the architecture of the NS2B/NS3 active-site interface [37], these differences suggest that the two inhibitors induce distinct local adjustments in the cofactor region, potentially reflecting differences in how their linker regions are accommodated within the protease binding site.

### Distinct linker geometries control intramolecular stabilization in the bound state

**Fig. 4:**
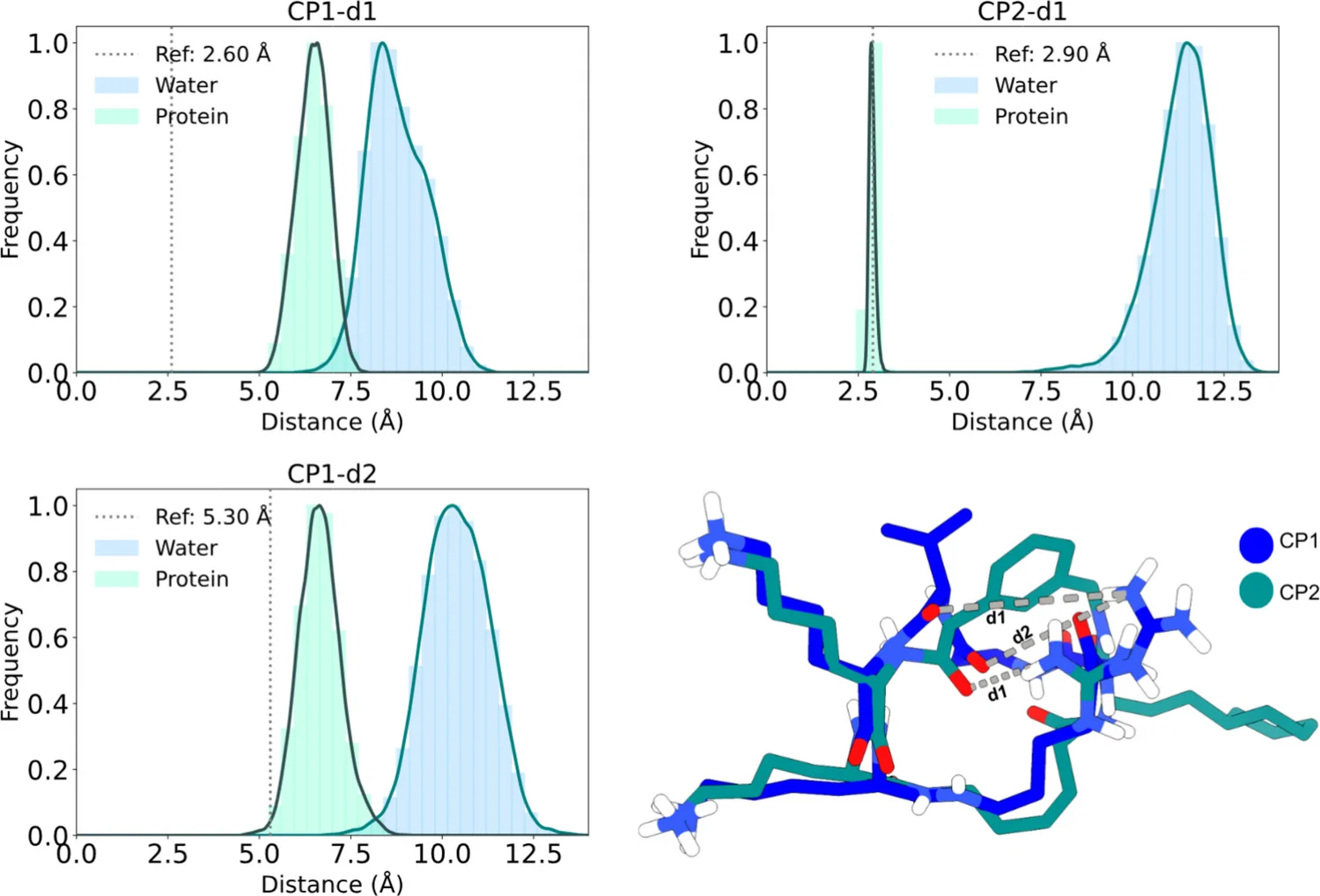
Intramolecular distance distributions for key internal contacts of the cyclic peptide inhibitors. Distance profiles monitored in aqueous solution and in the protease-bound state. CP2 shows more persistent short-distance contacts in the complex, consistent with greater bound-state preorganization, whereas CP1 retains a broader conformational distribution. The reference distances correspond to those observed in the crystallographic structure

Intramolecular distance analysis showed that CP2, but not CP1, preserves key crystallographic contacts upon binding to the ZIKV NS2B/NS3 protease (Fig. 4).

Distance d1 corresponds to the intramolecular hydrogen bond between the P4 carbonyl oxygen and the P1 guanidinium group, a contact described in the crystallographic complexes of this inhibitor scaffold. Distance d2 monitors an additional intramolecular contact involving a linker/backbone carbonyl located within the macrocyclic segment between P4 and P1, which can also orient toward the P1 guanidinium group and contribute to stabilization of the bound conformation. The orientation of these carbonyl groups is structurally relevant because it determines whether they can establish intramolecular polar contacts with the P1 guanidinium group. These contacts constrain the relative arrangement of the linker and the P1 region and therefore provide a direct measure of how the linker is organized within the macrocycle. Accordingly, the d1 and d2 distributions were used not simply as hydrogen-bond distances, but as local descriptors of linker-dependent macrocyclic organization.

The d1 and d2 distances were evaluated under two simulation conditions: the isolated peptide in aqueous solution and the fully solvated protein-peptide complex. In aqueous solution, both CP1 and CP2 displayed broad d1 and d2 distributions shifted toward larger values, with populations centered at approximately 11 Å (see Fig. S4). This indicates that these intramolecular contacts are not intrinsically preserved in the unbound state. In contrast, when CP2 was bound to the ZIKV NS2B/NS3 protease, the d1 distribution collapsed into a narrow population centered near 2.9 Å, closely matching the crystallographic distance. This suggests that the protein environment stabilizes a specific bound-state conformation in which the P4 carbonyl oxygen is properly oriented toward the P1 guanidinium group, thereby reinforcing the macrocyclic geometry of CP2.

For CP1, the crystallographic intramolecular contact corresponds instead to d2, with a distance of 2.6 Å. However, this interaction was not maintained during the MD simulations of the solvated protein-peptide complex. Rather, the d2 distribution shifted toward longer distances, with a maximum around 6 Å, indicating loss of the crystallographic contact in the dynamic bound ensemble. The corresponding d1 distance was also broader in CP1 than in CP2, suggesting a less defined orientation of the P4 carbonyl relative to the P1 guanidinium group. This broader distribution may be associated with the bulky hydrophobic P4 side chain of CP1, which, according to the crystallographic analysis of the original series, can alter the linker backbone conformation through close contacts with hydrophobic residues such as V155.

The comparison was further complemented by ligand RMSD and clustering analyses performed for both simulation environments (Figs. S4 and S5 and Table S1). Representative structures from the most populated clusters illustrate that both peptides undergo substantial rearrangement relative to their crystallographic conformations when simulated in water. In the protease-bound state, the protein environment restricts these conformational changes, although the two peptides retain distinct local arrangements around the linker and P1 guanidinium group. Thus, the global clustering and RMSD analyses support the local d1 and d2 distance profiles by showing that the aqueous and protein-bound environments produce different conformational ensembles (Fig. S4).

### Residue-level interactions and binding free energy support stronger recognition of inhibitor 2

**Fig. 5:**
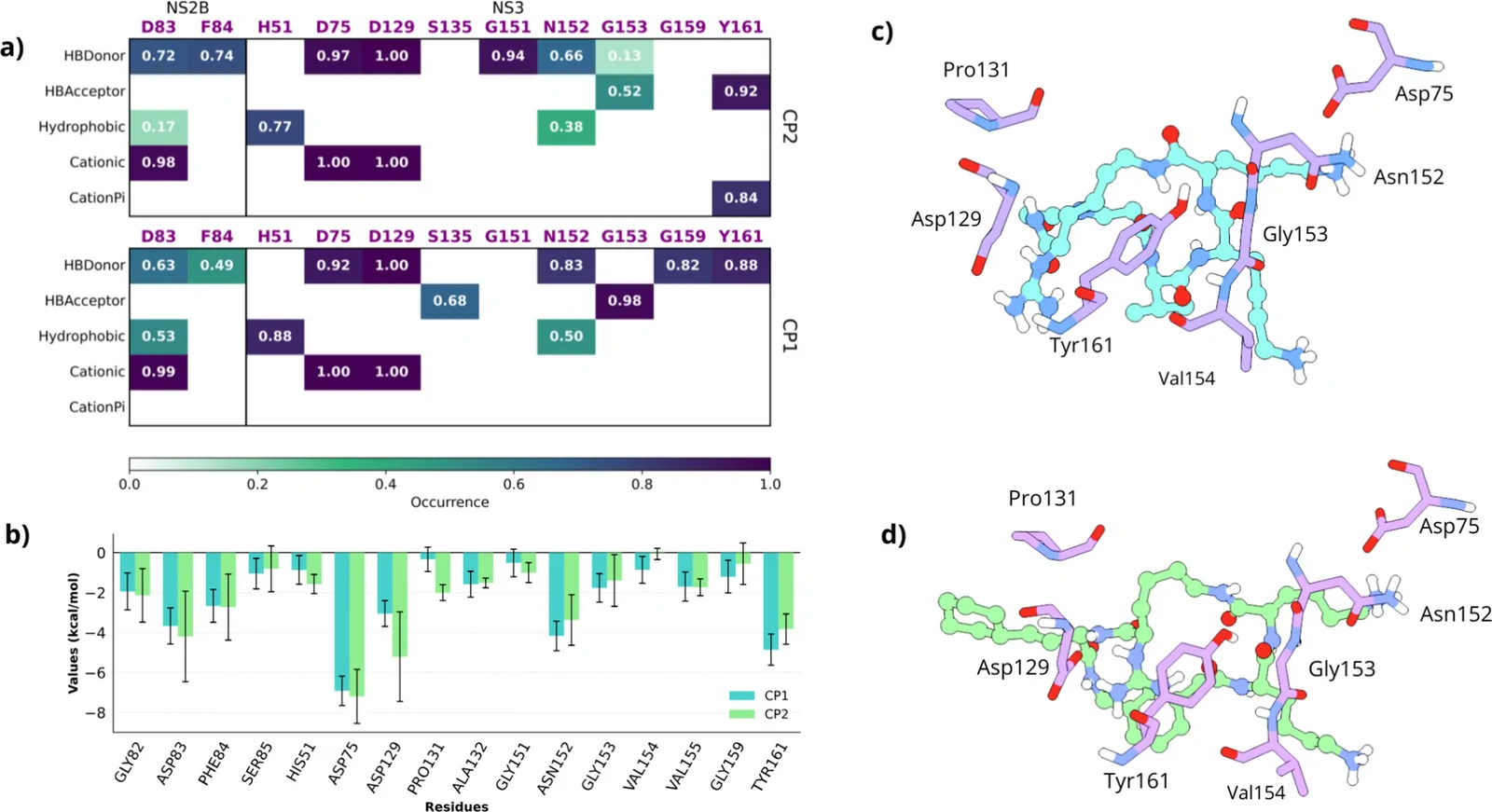
Residue-level interactions of CP1 and CP2 with the ZIKV NS2B/NS3 protease. **a** Interaction-occurrence maps for residues surrounding the binding site; **b** per-residue energetic decomposition, presented as the mean ± standard deviation across the three independent replicas; and representative binding-site arrangements for **c** CP1 and **d** CP2

The residue-level interaction analysis showed that both inhibitors are stabilized by contacts with residues located around the NS2B/NS3 active-site cleft, including Asp75, Asp129, Asn152, Gly153, Val154, Gly159, and Tyr161 (Fig. 5).

However, the interaction pattern differed between the two complexes. CP2 displayed a more focused and favorable interaction network involving Asp75, Asp129, and His51, suggesting closer engagement with residues associated with the catalytic and substrate-recognition region of the protease. The interaction fingerprint also showed persistent hydrogen-bond donor and cationic contacts with acidic residues, particularly Asp75 and Asp129, indicating that electrostatic anchoring plays an important role in stabilizing this ligand within the binding site. In contrast, CP1 showed a broader and more dispersed interaction profile. Although it also maintained favorable contacts with Asp75 and Asp129, its stabilization depended more strongly on residues such as Asn152, Gly153, Val154, Gly159, and Tyr161. This suggests that inhibitor 1 remains bound within the active-site groove but relies more on distributed contacts along the NS3 pocket rather than on a compact interaction network centered on the catalytic region.

The binding free-energy calculations are consistent with the residue-level interaction profile. CP2 showed more favorable binding energies than CP1 using both MM/PBSA and MM/GBSA approaches. The PB mean energy was −34.8 kcal mol^−1^ for CP2 compared with −27.6 kcal mol^−1^ for CP1, while the GB mean energy was −83.3 kcal mol^−1^ for CP2 and −69.8 kcal mol^−1^ for CP1. This corresponds to a more favorable calculated binding profile for CP2 by approximately 7.2 kcal mol^−1^ in MM/PBSA and 13.5 kcal mol^−1^ in MM/GBSA. The experimental binding free energies follow the same trend, with Δ*G*_exp_ values of −11.6 kcal mol^−1^ for CP2 and −8.7 kcal mol^−1^ for CP1 [15]. Inclusion of the normal-mode entropic contribution reduced the magnitude of the calculated MM/GBSA binding free energies for both complexes (Table S2). Nevertheless, CP2 retained a more favorable entropy-corrected binding free energy than CP1. Thus, the entropic correction did not alter the relative energetic ranking of the two inhibitors. The absolute calculated values remained substantially more negative than the experimental Δ*G* values, emphasizing that the end-point calculations and normal-mode estimates should be interpreted comparatively rather than as direct quantitative predictions of experimental binding free energies.

Computational alanine scanning provided an additional assessment of the contribution of selected binding-site residues (Fig. S6). Replacement of Asp75 and Asp129 by alanine produced among the largest positive ΔΔ*G* values in both complexes, indicating that the acidic side chains of these residues contribute favorably to peptide recognition. The mean effect of the Asp129 mutation was larger for CP2, consistent with its persistent cationic contact with this residue. Tyr161, Asn152, and Asp83 of NS2B showed moderate contributions, whereas the Val154 substitution produced a comparatively small energetic effect.

Although the absolute values from end-point calculations differ from the experimental estimates, all energetic descriptors consistently indicate stronger binding for CP2. Together, these results suggest that CP2 achieves more effective recognition of the NS2B/NS3 active site by combining a focused residue-level interaction network with more favorable binding free energy, whereas CP1 appears to compensate for weaker focused stabilization through broader contacts across the NS3 binding groove. These observations suggest that future linker optimization should consider not only direct protein contacts but also the ability of the linker to promote a favorable bound-state conformational organization and energetic profile.

## Conclusions

This study investigated the conformational behavior of two cyclic peptide inhibitors of the ZIKV NS2B/NS3 protease using molecular dynamics simulations and binding free-energy analyses. Although CP1 and CP2 share a common macrocyclic scaffold, their linker-dependent structural features led to distinct dynamic responses in aqueous solution and in the protein-bound state. In solution, both peptides displayed broad intramolecular distance distributions, indicating that the crystallographic contacts are not intrinsically preserved in the unbound state. Upon binding to the protease, CP2 more effectively preserved a short intramolecular contact compatible with the crystallographic d1 interaction, whereas CP1 lost its crystallographic d2 contact and sampled a broader bound-state ensemble.

Overall, the results suggest that the NS2B/NS3 binding site can stabilize specific macrocyclic conformations, but this effect depends on the orientation and steric properties of the linker region. CP2 showed a more defined bound-state organization, supported by persistent intramolecular stabilization, favorable residue-level interactions, and more favorable MM/PBSA and MM/GBSA estimates. While these simulations do not establish a direct causal relationship between individual contacts and inhibitory potency, they provide a structural rationale for how linker-dependent carbonyl orientation and bound-state preorganization may contribute to the stabilization of cyclic peptide inhibitors targeting flaviviral proteases.

## Supplementary Information

Below is the link to the electronic supplementary material.

ESM 1 DOCX (5.99 MB)

## Data Availability

The crystal structures used as starting models for the molecular dynamics simulations are publicly available in the Protein Data Bank under accession codes 7PFY and 7PGC. The molecular dynamics trajectories and analysis files generated during the current study are available from the corresponding author upon reasonable request.
