# Supplementary material for: Comparative molecular dynamics of two cyclic peptide inhibitors bound to the Zika virus NS2B/NS3 protease
Source: J Mol Model. 2026 Sep 7;32(10):349. doi: 10.1007/s00894-026-06945-8 (PMC13550072; doi:10.1007/s00894-026-06945-8)
Supplement: Supplementary file 1 [file 894_2026_6945_MOESM1_ESM.docx]

**Comparative Molecular Dynamics of Two Cyclic Peptide Inhibitors Bound to the Zika Virus NS2B/NS3 Protease**

*Camilla Vitoria Silva Marinho^a^, Maycon Vinicius Damasceno de Oliveira^a^ e Anderson H. Lima*^a^*

*^a^Laboratório de Planejamento e Desenvolvimento de Fármacos, Instituto de Ciências Exatas e Naturais, Universidade Federal do Pará, 66075-110, Belém, Pará, Brasil.*

*Corresponding author

**Camilla Vitoria Silva Marinho**

https://orcid.org/0000-0002-6162-3848

E-mail: camilla.marinho@icen.ufpa.br

**Maycon Vinicius Damasceno de Oliveira**

https://orcid.org/0000-0003-2263-2124

E-mail: maycondamasceno14@gmail.com

**Anderson H. Lima**

https://orcid.org/0000-0002-8451-9912

E-mail: [anderson@ufpa.br](mailto:anderson@ufpa.br)

**Fig. S1**

Root-mean-square deviation (RMSD) profiles of NS2B, NS3, and the cyclic peptides during the three independent 500 ns simulations of the CP1- and CP2-bound complexes. Trajectories were fitted to the initial minimized structure using the protease backbone atoms before calculation. Ligand RMSD was subsequently calculated without independent fitting of the cyclic peptide and therefore reflects deviations from the initial binding pose.

**Fig. S2**

Residue-wise root-mean-square fluctuation profiles of the ZIKV NS2B/NS3 protease over the three independent 500 ns simulations. RMSF values are shown separately for the NS2B and NS3 regions in the complexes containing (a) CP1 and (b) CP2.

**Fig. S3**

Representation of the protease surface, in purple corresponds to the NS3 protein while in gray the protein NS2B. a) CP1 e b) CP2

**Fig. S4**

 Representative structures of each cyclic peptide in water and in the protein, and the distances between the intramolecular hydrogen bonds formed in guanidine. a) CP1 and b) CP2

**Fig. S5**

Root Means Square Deviation for 500ns the simulation in water of molecular Dynamics for the three replicas independents

**Table S1.**

| System | (%) | Frames |
| --- | --- | --- |
| CP1_Protein | 34.9 | 52190 |
| CP1_Water | 52.0 | 77957 |
| CP2_Protein | 32.0 | 48020 |
| CP2_Water | 41.8 | 62643 |

Results of the clustering carried out over 1.5 μs of simulation for each simulated medium (water and protein), percentage and number of frames in the most populous cluster found, calculated using the k-means algorithm.

**Table S2.**

| Energetic quantity | CP1_mean | CP2_mean |
| --- | --- | --- |
| MM/GBSA ${\Delta G}_{bind}$ | -69.8±6.8 | -83.3±8.2 |
| ${T\Delta S}_{NMA}$ | -38.4±4.5 | -35.8±4.4 |
| Entropy-corrected ${\Delta G}_{bind}$ | -31.0±8.1 | -47,7±9.2 |
| SIE | -11.3±0.5 | -12.4±0.6 |
| Experimental ${\Delta G}_{bind}$ | -8.7 | -11.6 |

Binding free-energy estimates and normal-mode entropic contributions for the CP1 and CP2 complexes. All values are expressed in kcal mol^-1^ and reported as mean ± standard deviation across the three independent replicas. The entropy-corrected binding free energy was calculated as ${\Delta G}_{bind}$=${\Delta G}_{MM/GBSA}-{T\Delta S}_{NMA}$. Experimental binding free energies were derived from the reported *K*_i_ values.

**Fig. S6**

Computational alanine-scanning analysis of selected residues surrounding the CP1 and CP2 binding sites. $\Delta\Delta G_{Ala}$ was calculated as the difference between the binding energy of the alanine-mutated and wild-type systems. Positive values indicate destabilization following alanine substitution. Bars represent the mean values obtained from the three independent replicas, and error bars indicate the corresponding standard deviations.
